# Lived Experience of Fully Closed-Loop Insulin Delivery in Adults with Type 1 Diabetes

**DOI:** 10.1089/dia.2023.0394

**Published:** 2024-03-28

**Authors:** Rama Lakshman, Sara Hartnell, Julia Ware, Janet M. Allen, Malgorzata E. Wilinska, Munachiso Nwokolo, Mark L. Evans, Roman Hovorka, Charlotte K. Boughton

**Affiliations:** ^1^ Wellcome-MRC Institute of Metabolic Science, University of Cambridge, Cambridge, United Kingdom.; ^2^ Cambridge University Hospitals NHS Foundation Trust, Wolfson Diabetes and Endocrine Clinic, Cambridge, United Kingdom.; ^3^ Department of Paediatrics, University of Cambridge, Cambridge, United Kingdom.

**Keywords:** Closed-loop system, Type 1 diabetes, Qualitative, Psychosocial

## Abstract

**Introduction:**

The Closing the Loop in Adults With Type 1 Diabetes (CLEAR) randomized crossover study compared a novel fully closed-loop insulin delivery system with no carbohydrate entry or mealtime bolusing (CamAPS HX), with standard insulin pump therapy and glucose sensor in adults with type 1 diabetes and suboptimal glycemic outcomes. This qualitative substudy aimed to understand the psychosocial impact of using the fully automated system.

**Materials and Methods:**

Adults participating in the CLEAR study were invited to take part in a virtual semistructured interview after they had completed 8 weeks using the fully closed-loop system. Recruitment continued until there was adequate representation and data saturation occurred. Interviews were anonymized and transcribed for in-depth thematic analysis using an inductive-deductive approach. Study participants were also asked to complete questionnaires assessing diabetes distress, hypoglycemia confidence, and closed-loop treatment satisfaction.

**Results:**

Eleven participants (eight male and three female; age range 26–66 years) were interviewed. After an initial adjustment period, interviewees reported enjoying a reduction in diabetes burden, freed-up mental capacity, and improved mood. All were happy with overnight glycemic outcomes, with the majority reporting benefits on sleep. Although experiences of postprandial glucose outcomes varied, all found mealtimes easier and less stressful, particularly when eating out. Negatives raised by participants predominantly related to the insulin pump hardware, but some also reported increased snacking and challenges around resuming carbohydrate counting at trial closeout.

**Conclusions:**

In adults with type 1 diabetes, use of a fully closed-loop insulin delivery system had significant quality-of-life benefits and provided a welcome break from the day-to-day demands of living with diabetes.

**Clinical Trial Registration:**

NCT04977908.

## Introduction

Intensive insulin therapy represents a significant management burden for people with type 1 diabetes, contributing to burnout, diabetes distress, and reduced quality of life.^1^ Hybrid closed-loop insulin delivery systems, which automatically calculate and adjust insulin rates, are transforming the management of type 1 diabetes. They have been shown to improve both glycemic outcomes^2^ and quality of life.^3^ However, all current commercially available systems still require users to count carbohydrates and manually input insulin boluses before meals and snacks.

Accurate carbohydrate counting can be challenging and adds significantly to the burden of day-to-day diabetes management.^4,5^ It can negatively affect quality of life, making people with type 1 diabetes feel restricted in food choices and more socially anxious among peers.^6^

Newer ultrarapid insulins are now available, which make a fully closed-loop approach, without the need for carbohydrate counting or premeal bolusing, feasible. We recently reported significantly improved glucose outcomes without increasing hypoglycemia when using a novel fully closed-loop system (CamAPS HX) with ultrarapid insulin lispro (Lyumjev) compared with insulin pump therapy with a glucose sensor in adults with type 1 diabetes and suboptimal glycemic outcomes (glycated hemoglobin [HbA1c] ≥8.0%).^7^

To complement the glycemic data, this psychosocial substudy used semistructured interviews and validated questionnaires to understand the lived experience of using the fully closed-loop system. There have been no previous qualitative evaluations of fully automated insulin delivery systems, and this is an important aspect of research in this field, with quality-of-life impacts of diabetes technology often being as important to users as glycemic benefits.^3^

## Materials and Methods

We interviewed and collected questionnaire data from participants who took part in the CLEAR study. This was a single-center randomized crossover trial involving 26 adults with type 1 diabetes using insulin pump therapy and with a glycated hemoglobin ≥8.0% (64 mmol/mol) at baseline.^7^ In the CLEAR trial, participants were randomized to 8-week use of a fully closed loop system (CamAPS HX; CamDiab, Cambridge, UK) with Lyumjev insulin (Lilly UK, Basingstoke, UK) or 8 weeks of their usual insulin pump therapy with glucose sensor, before crossing over to the other arm.

The CamAPS HX system comprised a control algorithm residing on an app on an unlocked Android smartphone, receiving sensor glucose data from a Dexcom G6 glucose sensor (Dexcom, San Diego) and directing insulin delivery on a Dana RS insulin pump (Sooil, Seoul, South Korea). During the fully closed-loop period, participants were advised not to announce, enter carbohydrates or bolus for any meals or snacks. Users could adjust the glucose target on the app (customizable between 4.4 and 11.0 mmol/L), and also use the “Boost” and “Ease Off” function on the app to temporarily increase or decrease insulin delivery. There was a bolus calculator available on the app that participants were trained to use for safety.

### Recruitment and data collection

On completion of the study, participants were invited to take part in a virtual in-depth interview through Zoom (Zoom, San Jose, California) at a time of their choosing. Interviews averaged 40 min and were conducted by a researcher trained in qualitative research methods. The researcher was not involved in the clinical care of trial participants and participants were reassured of confidentiality and encouraged to share negative experiences as relevant.

Interviews were informed by a topic guide (Table 1), to ensure the key study objectives were covered, while also allowing participants to raise themes they considered important. The topic guide was developed based on literature review, input from patient representatives and members of the clinical team and was revised in light of emergent findings. Recruitment continued until there was adequate representation in terms of age, gender, and educational status and data saturation had occurred.

**Table 1.** Topic Guide

Previous experiences of diabetes technology Motivations for taking part in the trial and initial expectations Ease of use of study equipment Initial experiences of using the fully automated system, including adjusting to not announcing meals/bolusing If and when confidence and trust in the system were established Impact of using the fully automated system on the following: Dietary choices Sleep Physical activity Work Social and family life Impact of system on the practical and mental burden of diabetes management Views on trial closeout, and going back to standard insulin pump therapy Views about how the system could be improved to aid efficacy and acceptability Views on which individuals would most benefit from using a fully closed-loop system^a^

^a^ Topic added in light of interviewees suggesting that certain characteristics of their lifestyle made the system particularly helpful.

Two validated questionnaires were given to all participants on completion of both study periods to evaluate hypoglycemia confidence^8^ and diabetes distress.^9^ An additional two questionnaires, a validated questionnaire to evaluate closed-loop treatment satisfaction,^10^ and a “closed-loop experience” questionnaire were administered to participants on completion of the fully closed-loop period. Participants were asked to complete the questionnaires at home and return them to the research team.

### Data analysis

Interviews were transcribed in full, anonymized, and then analyzed thematically using an inductive-deductive approach. Interview transcripts were reviewed and cross-compared with look for recurrent themes. Four members of the research team were involved in data analysis, and together developed a coding framework to capture all key themes. The qualitative analysis software package NVivo 12 (QSR International, Doncaster, Australia) was used to facilitate data coding and retrieval. We followed the Standards for Reporting Qualitative Research.^11^

Analyses of questionnaire data were carried out using SPSS Statistics software, version 28 (IBM Software, Hampshire, UK).

### Ethics

Before commencement, approval for the CLEAR study and interview substudy was received from an independent research ethics committee in the United Kingdom.

## Results

Eleven participants were interviewed, demographics are shown in Table 2. The key themes cut across the data set, so we have not separated reporting according to individual characteristics such as gender or age; however, these details are reported after each participant quotation.

**Table 2.** Demographic Characteristics of Participants Taking Part in Interviews

Characteristic	
Age (years)	41.5 ± 12.7 (range 26–66)
Gender—female, *n* (%)	3 (27)
Race/ethnicity, *n* (%)	
White European	9 (82)
White other	1 (9)
Asian	1 (9)
Education, *n* (%)	
University	7 (64)
College	2 (18)
School	2 (18)
Duration of diabetes (years)	19.9 ± 9.8 (range 5–45)
Duration of pump use (years)	8.0 ± 3.8 (range 2–13)
Previous CGM use, *n* (%)	6 (55)
HbA1c at baseline (%)	8.8 ± 0.6 (range 8.1–9.8)
HbA1c at baseline (mmol/mol)	73 ± 7 (range 65–84)

Data are mean ± SD, *n* = 11.

CGM, continuous glucose monitoring; HbA1, glycated hemoglobin; SD, standard deviation.

### Initial thoughts: excitement, expectations, and adjustment

When discussing their motivations for taking part in the trial, all interviewees stated altruistic reasons, wanting to contribute to “anything that would benefit others in the future” (2_M_55Y). There was also overwhelmingly a sense of excitement about fully closed loop, that “it was incredible to be part of the future of diabetes” (9_M_33Y). For example, one participant described the first time not-bolusing for a meal: “That's when all the feelings kick in of this is genuinely here now. We've always said oh ten years, ten years, ten years. It's like it's happening now. It was not overwhelming, I guess. But it was like a moment. That first time” (10_M_28Y).

Although some interviewees described extremely high hopes of the study system going in “before I tried out, I was thinking sort of great, I'm normal for a while” (3_M_53Y), the majority were “a bit apprehensive at first” (4_M_32Y). Several described considerable worry about handing over full control to the closed-loop system: “Especially with the diabetes, especially if you've had it for a long period of time, you think you're the only person who can control it. And even though you might not be controlling it well, you don't want to give that control up” (9_M_33Y).

Reassuringly, all interviewees did find they were able hand over control and trust the fully closed-loop system by the end of the 8-week period. A few participants found the change very easy “well of course not having to do something is easier to adjust to than having to do something” (11_M_46Y) however, the majority described a transition period during which they had to adjust to a “whole new way of thinking and doing” (3_M_53Y).

This generally lasted from a few days to a couple of weeks: “It was a bit odd initially. I kept looking at the pump and I was also checking manually. After one or two days I was like, oh, I'm very impressed with the technology…. And then I didn't have to do anything. I didn't have to worry because I knew the pump was doing the right thing” (1_F_42Y).

Further quotations on participants' initial thoughts and adjustment to the fully closed-loop system are captured in Table 3.

**Table 3.** Initial Thoughts on the Fully Closed-Loop System

Theme	Participant quotations
Excitement about fully closed loop	I could see it was a complete closed-loop, working like your own pancreas (5_F_66Y). So when I was first diagnosed… they said, look in the next 15 to 20 years there'll be a more artificial form of pancreas. Which is pretty much what this is in my opinion. So just the sheer fact that I got to see this live and be part of it, it was nothing but excitement to be honest (9_M_33Y). And then just the sheer ingeniousness of having, not a hybrid closed-loop but an actual closed-loop system is obviously the way forward isn't it? It's what it probably will look like in five years or so. So just taste of the future (11_M_46Y).
Expectations and anxieties going in	I remember [the study clinician] did say that this is not a magic wand that can fix and create a life back to as it was pre-diabetes. And I'm a little bit of a worrier at heart. And it did come across possibly as something that was a stab in the dark (2_M_55Y). I didn't think it would have been as good as it was. Cause obviously never trying it before I hadn't put a lot of faith into it that it would work, and obviously it did work really well (8_M_26Y). It's like well now I'm going to trust a little app on a phone and the pump to do all this work for me. So there's definitely a level of is this going to work or is this going to bomb out (9_M_33Y). And I was a little bit skeptical as well. Just how good can it be? Cause it was very easy to run away with it in your head as to this being the answer to everything (10_M_28Y).
Adjustment and trusting system	Yeah, it's like anything new, you always want to keep testing it and yeah, checking it and checking it… But I mean the study was long enough for me to eventually put it to one side and say, right, okay, that's a week, 10 days done. And we are okay (2_M_55Y) Very, very sort of weird. So just not having to count the carbs and think of what you're doing that day and at that time it's just, yeah, it took some getting used to … probably a couple of weeks I would say (3_M_53Y). I just sort of went in from day one, like okay I don't need to bolus ever again now for next [two] months. But as a backup I carried my [usual] pump on me for the first couple of weeks just in case I was having a period of time where I just thought, you know what, I can't trust it. But it never came to that. It always worked without any issues (4_M_32Y). It definitely took me at least a week I think to comprehend that I need to step back and let this do what it's been built to do. And then slowly over time I eased more into and more into it. But the first week is really when I really had to sit there and think, well I've got to trust it, because if I keep trying to make changes, it's not doing its job and it's not going to be effective (9_M_33Y). I wanted to really just give my trust in it straight away and then take advantage of it for as long as I could really (10_M_28Y). With any new gadget, you get a bit obsessed with it for the first two or three weeks don't you… I suppose once it settles down, it's less time [thinking about diabetes] cause you're not having to carb count (11_M_46Y)

### A break from diabetes without compromising control

#### A break from diabetes

A universal theme was that the fully closed-loop system represented a welcome break from the day-to-day demands of living with diabetes: “I did not have to do anything. Which was fantastic. And it just made my life so so much easier… I just felt I was on a holiday when I was on that.” (1_F_42Y). “Obviously the fully closed loop it is literally you can live your normal life if you know what I mean. You can just cook what you want for dinner, eat it and sit down and not have to work everything out. Because it picks up and sorts itself out… It was just nice to have a little break” (8_M_26Y)

Although some participants did reference ongoing user responsibilities relating to insulin pump therapy, these were invariably presented as minimal compared with what they were doing before: “Oh it was liberating actually. I didn't have to think, I didn't have to look at a plate and count. I didn't have to check my blood glucose levels…I just genuinely felt like I was a non-diabetic. I could do whatever I wanted when I wanted and know that I was within control. I guess the only consideration was how much insulin is left in the vial, do I need to take a backup or whatever. But other than that.” (6_M_44Y).

#### Satisfactory glucose outcomes

Importantly, this “holiday” from the demands of diabetes came without a perception of compromising glucose outcomes. Although not perfect, almost all participants reported subjective improvements in glucose levels. For some participants the improvement was dramatic: “The one thing I noticed was my blood sugars were completely under control” (1_F_42Y).

Several pointed out the beneficial effect of the system learning over time: “I thought the algorithm, for me particularly sort of six weeks in was absolutely fantastic. Looking at my results from what I can remember, I didn't have a massive deviation between low and high. It was pretty flat all day, with the exception of when you have your main meals you peak a little bit, but those peaks were nowhere near as high as what they would've been previously (4_M_32Y).”

For others, postprandial hyperglycemia was more of an issue, but in general participants described how “sustained highs, [were] lot less on the trial” (10_M_28Y). They also described how the high levels after eating became less of a problem when overnight control was invariably good. “So yes, there was a little bit of an increase [post meals], but then the thing that is most important to remember is that come seven o'clock in the morning when I get up and get going, I'm in target” (2_M_55Y). However, one participant did report a perceived negative effect on overall glucose outcomes, saying that the Lyumjev insulin “just didn't work quick enough. So more time than not, I was high.” (3_M_53Y).

#### Hypoglycemia

Fear of hypoglycemia was a theme in more than half of interviews. For example, one participant explained he “often ran higher so I've avoided hypos because I really don't enjoy having them,” and in this context “had a lot more hypos on the [fully closed-loop system] than I did normally” (10_M_28Y).

However, for the majority of participants, including those who previously ran high to avoid hypos, the hypoglycemia burden was similar or lower while on the fully closed-loop system: “I do get very nervous around hypos. So if I see my sugar pushing low and I know I'm physically busy, I will actually have a coke or I will have something to try pre-empt that low… I would say I was less worried about lows on the system. Quite simply because it just has that cut-off [for insulin delivery] where the closed loop is already doing its job by the time you start thinking about it or doing it. So it's quite great” (9_M_33Y).

“I used to worry, especially before driving, before going to exercise, and if I don't eat a meal for a couple of hours… what should I do, should I check my blood sugar, should I eat something? I'm very cautious about these things. I don't want hypoglycemia and things like that. But with the pump I didn't have to worry” (1_F_42Y).

Two participants reported a transient period of increased hypoglycemia when starting the fully closed-loop system: “I think at the beginning, probably more frequently than before I was on the study because it was getting used to me. But I would say within a couple of weeks my level of hypos was significantly lower than normal” (6_M_44Y).

Further participant quotes relating to these themes can be seen in Table 4.

**Table 4.** A Break from Diabetes Without Compromising Glucose Control

Theme	Participant quotations
A break from diabetes	I mean the freedom of it was lovely. Just being able to eat and not have to worry, count carbohydrates and yeah, the freedom it gave was brilliant (2_M_55Y). Yeah, it was just a sense of normality. Very minimal maintenance other than making sure that there's battery in the pump, making sure the reservoir was full (4_M_32Y). Just I could carry on my life. Well, I always carry on my life, you know, my diabetes doesn't interfere with me. So, yeah, so I just really carried on as normal, but it just made certain things a lot easier (5_F_66Y). It was great. I felt like the closest to the normal person, that a diabetic could be. It was not having to think I've got to work out how many carbs I'm eating, I've got to get my pump out, people are going to look… Just easy not having to think oh no, I haven't done my insulin (7_F_32Y). It was almost like a little bit of a break in terms of the bolusing and that…Once you start seeing it working and doing what it should, you definitely start going, okay, well this is actually great. This takes out quite a bit of the calculation and all of that. Less mental strain almost. It didn't catch everything and sometimes you do need to make a few amendments, which I don't think is a big ask, but for a lot of it, it is quite automated (9_M_33Y). It was quite liberating I think…taking that mental burden that I've often had because I weigh all my carbs, make sure every meal and kind of sum up the total in your head of all these different little things. Removing all of that was like okay, no hybrid system could ever come close to doing that (10_M_28Y). You're not having to carb count you're not having to constantly evaluate everything you eat or do. Yeah, really nice just to take a break from diabetes almost for a bit (11_M_46Y).
Satisfactory glucose control	It started to learn what I was doing and it responded quite quickly to that. It did work well… whatever I ate, it was fine. I mean, the only time if I did have, and I don't often have, a pudding.… It maybe shot up a little bit, but it then just leveled off and dropped again. So it actually coped with that and then came down. And overnight it was great. It stayed lovely, steady line all the time (5_F_66Y). I mean the level of hypos I suffered and the level of high hypers was very minimal actually, I was very much in range (6_M_44Y). Overnight they're absolutely perfect figures. When you look back on the graph it cut in, cut off and just did what it should really. So yeah, it was good (8_M_26Y). So I think for what it is, it did really well. It's not a hundred percent at catching the lows and the highs, and I think over time it that will improve with technology and just simply, I think more time on it makes it better for you (9_M_33Y). I know by the morning I'm going to be perfectly normal blood sugar, everything's going to be fine… And then you still win the day (10_M_28Y). I suppose I was still getting spikes because with the best will in in the world, even with the Lyumjev insulin, it wasn't going to keep up with breakfast particularly (11_M_46Y).
Hypoglycemia	So yes, I mean I just cranked [the personal glucose target] up until I was just getting occasional hypos. So I think I went back to 7 and then down to about 6.5 is kind of where I settled on in the end (11_M_46Y).

### Improved quality of life: greater flexibility around food, improved sleep and productivity

#### Improved quality of life

Another key theme reported by interviewees was that reduced practical burden of diabetes was associated with improved mood. As one participant put it: “So burnout is a real thing. You don't realise it until actually a chance to step back and realise actually wow” (11_M_46Y). Participants described feeling “happier and much more relaxed” (1_F_42Y) and described how this improved mood also had “a positive impact on the people around [them]” because “The diabetes can be super stressful, so if the system is taking off any of the load, then it is relieving something off my load. It ultimately makes me a less grumpy person to be honest” (9_M_33Y).

#### Greater freedom around food

The biggest quality-of-life impact appeared to be around eating out with one participant explaining “Very easy and very free. Takes a lot of the stress and worry, especially when you are going out and eating, the actual concept of it is brilliant” (3_M_53Y). Again, this benefit extended beyond the user themselves “being able to just [go out and eat] and not worry about it… I didn't feel like I had to say no because oh we've eaten out three times this week and I've had really bad blood sugar control for the whole week as a result. So, eating out is obviously an important thing for my partner, so to be able to do that more frequently was good for us” (10_M_28Y).

Most participants reported that they “didn't at any point feel like [the closed-loop system] was limiting [them] or changing [their] meal choices” (4_M_32Y), and indeed provided “a bit more freedom to eat when [they] wanted without having to worry about the impact” (6_M_44Y). One participant actually reported a negative impact of this flexibility “Psychologically, the fact that I knew all of a sudden, I could eat the things that I wanted to eat without thinking about it… made me tend towards more junkie food. I think that there is an interesting risk in a way of falling into that trap of now not looking after yourself as well as you could. Because suddenly you don't have to make all these decisions which do put you into a position of making better choices” (10_M_28Y).

However, another participant reported snacking more, but on healthier foods and yet another, who was new to glucose sensors, reported looking at blood glucose levels made her “very careful what [she] was eating, which is a good thing” (1_F_42Y).

#### Sleep

The majority of participants reported improvements in sleep while on the system, secondary to reduced hyperglycemia; one participant who previously woke up six nights a week described how “without the overnight high you sleep right through. So, I could actually sleep for the time I was on the [fully closed-loop] system” (8_M_26Y). Another reported “I felt better in the morning because I hadn't had to get up at four o'clock to go to the toilet. I wasn't feeling lethargic” (2_M_55Y). In contrast, three participants did report “waking up in the night [due to] getting the low glucose alarm” (4_M_32Y), but in two cases this was resolved by increasing the personal glucose target overnight.

#### Daytime productivity

Another finding from more than half the interviewees was that using the system had positive impacts on work and productivity. One participant, who has a physically active job, described that with the automation it was “quite comforting to know that, okay, I could take my hands off the wheel a bit and I could focus on the job and not have to constantly think, what is my sugar doing right now” (9_M_33Y). This was also true beyond work, another participant described: “My output in general has increased a lot. Not having all the decisions that I normally have to make. And I did find that I was doing more in the evenings and exploring more of my hobbies in the evenings and doing new things. Cause yeah, I just wasn't using all that brain power all the time on this one thing.” (10_M_28Y).

However, this only came after the initial period of algorithm adaptation and users developing trust in the system. One participant described how “for that first two weeks when it was kind of learning me and my behaviours and my blood glucose levels I would've deliberately not done as much client facing or putting myself in an environment where a hypo would've been an inconvenience” (6_M_44Y).

Other quotations relating to quality-of-life impacts are outlined in Table 5.

**Table 5.** Improved Quality of Life

Theme	Participant quotations
Improved mood and less worry	Took the worry from me and also the worry from my wife as well. Cause she wasn't worried about me going low or going too high (3_M_53Y). I think my husband would have told you I was happier because, I said to him it felt a holiday from diabetes (7_F_32Y). And I think just my wellbeing in general has improved until sort of the last couple of weeks and you start realizing it's coming to an end and then it sort of goes the other way. But yeah, I definitely had a hugely improved mood I would say (10_M_28Y).
Greater freedom around food	In terms of quality of life, I would say yeah, definitely massively improved quality of life, particularly eating out because carb counting can be that little bit harder when you're eating out (4_M_32Y). I would have days where I would just have salads and no carbs at all. I had days where, I'd eat half a cake and then binge eat you know a packet of biscuits, carton of orange juice to really, really test the algorithm and see how it works (4_M_32Y). Well in a sense it liberated me to not have to worry about it so much. Cause I don't know if I fancy a biscuit now, if my blood sugars were 10, I would just not have that biscuit because I know it would cause problems. Whereas with the closed loop system, I wouldn't ever have sugars above seven, maybe eight if I'd just eaten (6_M_44Y). I remember loving the fact I didn't have to count and just felt a bit freer [around food] I suppose (7_F_32Y). It definitely in my opinion, allowed a bit more flexibility (9_M_33Y). So was I more prepared to take small snacks if you see what I mean? You're not constantly having to put in a bolus for them. And yes, I probably did to be honest. So I mean the flip side of that is smaller snacks probably mean healthier snacks for me (11_M_46Y)
Sleep	I wasn't waking up so tired in the morning. Because some mornings {prior to the study] I was waking up thinking why am I 16, 17? Why are my levels so high? How is that even possible? … But the algorithm adjusting in accordance with your body's reactions. Yeah, it's brilliant (4_M_32Y). Going back to the point that my blood glucose were pretty much always in range. So I slept well, possibly deeper (6_M_44Y). With high sugars as you get up to go to the loo in the night, so definitely not doing that. Obviously lows in the middle of the night is a pain but actually after putting up the personal target and getting rid of the lows my sugars were still consistently below 10. So yeah, slept well, slept better (11_M_46Y)
Daytime productivity	I do remember not working too far away from the house for about the first month so that I could rush inside and get some as sustenance if required (2_M_55Y). There's nothing worse than bolusing and then get called off somewhere [to an emergency] and having to not eat. So not having to worry about that was great (11_M_46Y).

### System-specific features: appreciated opportunities for modulating insulin delivery, disliked hardware

#### Modulating insulin delivery

All users found options to modulate insulin delivery through the “Boost” and “Ease Off” function helpful. Boost was used variably from every day to just once during the 8-week period, but most participants described using it occasionally if “feeling unwell or if [they'd] had something that was very high GI or very high in fat” (4_M_32Y). They also described benefits when eating out: “If you know you're going to be eating a lot of carbs hit the boost when you've ordered your food. That did bring it all down and keep it down” (8_M_26Y).

The “Ease-Off” function was primarily used for exercise, but also found useful by those who had active jobs: “If I'm on my way to a job where I know I'm going to be busy for a few hours, I'll ease off, because I know generally that's when I'm going to push low” (9_M_33). Although most participants liked the simplicity of “just pressing a button to make the algorithm more or less aggressive” (9_M_33), one user indicated they would have liked more prescriptive guidance on “how long to Boost or Ease Off for certain things” (10_M_28Y).

#### Hardware

Almost all the negative comments about the fully closed-loop system were concerning the study hardware. There was a strong consensus that “the worst thing about all the technology was the pump” (2_M_55Y). Participants found that compared with the pumps they had used previously “it was quite clunky” and “quite a faff changing the insulin every time” (7_F_32Y).

Another common issue reported was the “device-specific battery” that “failed quite a number of times without warning” (4_M_32Y). Participants liked the CamAPS HX app, which was “really user friendly, it was very visual” (4_M_32Y); however, most had to carry a separate study phone as the app is currently not iOS compatible. More than half of the participants reported that “having to carry around two phones is a bit of an annoyance” (11_M_46Y).

### Trial closeout: initial difficulties going back to bolusing, interest in continued use of fully closed loop, and ideas for improvement

#### Back to bolusing

At trial closeout, most participants struggled with going back to carbohydrate counting and bolusing. As one user described “I probably got a little bit lazy in my actual carbohydrate counting because the machine was doing it for me” (2_M_55Y). For almost all participants these skills came back within a few days; however, one participant reported that several months after completing the study “even now I sometimes forget to bolus, whereas [with fully closed loop] it wasn't a fault… So I adjusted really well to that idea, but it was hard adjusting back to normal normality” (9_M_33Y).

#### Future use of fully closed loop

Participants all expressed interest in using the fully closed-loop system if it were commercially available, saying “I was quite enjoying it at the end – it stopped too soon” (5_F_66Y) and “I'd love to stay on it forever” (7_F_32Y). Interestingly, five participants went on to use a hybrid closed-loop system after finishing the study and four of these preferred the fully closed-loop system: “So being on the fully automated system, it worked far better than being on a hybrid closed loop for me… I'd say to you, if it's available tomorrow I'll take it. Even over the hybrid, yeah definitely” (4_M_32Y).

Another described, “Don't get me wrong, I'm very happy with the [hybrid closed-loop system]. It does its job. It doesn't do the job that that system does. I do think that that system is a bit golden…. I do think it gave better control, less stressful. That system is literally like the dream” (9_M_33Y).

The most common drawback to using the system was the insulin pump, with one participant saying “I'd probably think twice around the hardware pieces. But I think the advantages outweigh that as a disadvantage” (6_M_44Y). Two participants suggested they would prefer a little more interaction with the system around mealtimes to help with postprandial glucose excursions: “Keep the idea about it essentially carb counting for you. But I would really like to tell it that I'm about to eat something sugary or a big meal or something with a significant amount of carbs just so it could be ahead of the game” (11_M_46Y).

The other participant similarly described they would like “the opportunity to be able to say, look, I'm about to have a big meal, give it a little bit more information to help it make better decisions” (10_M_28Y). However, he also described wanting to keep the full automation some of the time: “If I'm out for dinner, I don't know what I'm eating. And then that's where the magic of the algorithm comes in” or “where I can't be bothered to weigh my food… if you want a night off you can have a night off.”

#### Who could benefit

When asking participants who they thought would most benefit from the fully closed-loop system the themes were those who struggle with carbohydrate counting, but also those who had unpredictable lifestyles or were busy with priorities other than managing diabetes: “I think for me, because I've got quite an unpredictable lifestyle, that's where it works really well. Somebody who's active like me, sort of sporty does things like that, that's good. And people who just generally struggle with carb counting and control I would say would benefit too” (6_M_44Y).

“To be honest, I think people who are busy, it'll help a lot with, because as we've said, it kind of removes a part of the strain on your mind. It frees your mind up to deal with some other things” (9_M_33Y). And as one participant observed “yes, you should really make time for your diabetes because it's the one thing that's keeping you alive, but it would make life a lot easier for people who are totally beholden to running around for other people” (2_M_55Y).

Further quotes around trial closeout and future use of fully closed loop are reported in Table 6.

**Table 6.** Trial Closeout and Future Directions for Fully Closed Loop

Theme	Participant quotations
Difficulties in going back to bolusing	Now I have to think and now I have to enter everything on the pump, so going back to doing the same thing was a little bit difficult. I have to concentrate what I'm doing. Yeah. But it's fine. After a couple of days I was okay (1_F_42Y). It was a bit of a shock when I had to do all the carb counting and that again when I came off. It was hard for a while… Well, probably a few days just sort of remembering. I would eat something and think actually, I should have counted that carb and put that through my pump (3_M_53Y). To start with after being on a fully automated system? I did have several occasions where I completely forgot [to bolus]. Yeah, because it was almost abnormal to go back to something which had been so normal previously. But within a week I was bolusing at every single meal (4_M_32Y). [Carbohydrate counting] is kind of like second nature, so I got back into it. But yeah the first week it felt awful going back because you got used to the other system so quickly, the bolusing was a pain so having it do it for you was brilliant (7_F_32Y). Yes, it's rather peculiar the first week or two getting back into it I suppose. And I mean carb counting because I've done it for 17 years, something like that. So it comes back quite quickly. So it's a little bit odd for a short period but it's fine (11_M_46Y).
Eagerness to use fully closed loop in the future	What's going to happen is [the fully closed-loop system] going to come out? If in the future something comes up, then the one which now we are talking about would be the one I would go for (1_F_42Y). Oh, I would use I would choose the [fully] closed loop system {over the hybrid closed-loop system]. Just that it controlled my blood sugars better. Yeah, it just made my body glucose levels better and kept the levels correct really (5_F_66Y). If I got offered that system to use full-time, I would use it. I do think it was good and it just makes it a lot easier if you know what I mean. You'd, just rely on it, to do what it's going to do and it will do what it should (9_M_26Y).
Most benefits to those who are busy, or have an unpredictable lifestyle	It'll be beneficial for people like me who are young and who are busy with children, who's got one or two kids and I don't know when they have a busy life working and everything (1_F_42Y). I think for somebody who is extremely active, somebody who has a lot going on in their life, I think it is essential because I mean we've got two children under the age of 10 and it's hectic, absolutely hectic (02_M_55Y). So anyone with a lifestyle that isn't predictable I suppose. mean for shift work it's nice in some ways the whole idea of it correcting every 10 minutes is great. Then it completely screws up the idea of it learning your patterns. But it wasn't that it didn't cope… I suppose the flip side is anyone who can't do their own carb counting. So you know dementia, disability whatever. I mean you've got a ready-made market haven't you (11_M_46Y).

### Questionnaire results

Table 7 reports the results of the closed-loop experience questionnaire, which was returned by 19 study participants. Everyone was happy to have their glucose levels controlled automatically, 94% of responders would recommend closed loop to others, 89% spent less time managing their diabetes, and 78% slept better. Many of the themes identified in the free text of the questionnaire are similar to those identified in the interviews, including improved mood and reduction in burnout as well as the negatives raised around postprandial glucose excursions and the study pump.

**Table 7.** Responses to Closed-Loop Experience Questionnaire (*n* = 18)

	Strongly agree	Agree	Neutral	Disagree	Strongly disagree
Q1. I was happy to have my glucose levels controlled automatically by the system	14 (78)	4 (22)	0 (0)	0 (0)	0 (0)
Q2. I spent less time managing my diabetes	14 (78)	2 (11)	2 (11)	0 (0)	0 (0)
Q3. I was less worried about my glucose control	10 (55.6)	7 (39)	1 (6)	0 (0)	0 (0)
Q4. Using the system took more time and work than it is worth	1 (6)	1 (6)	1 (6)	2 (11)	13 (72)
Q5. I slept better during the nights	9 (50)	5 (28)	4 (22)	0 (0)	0 (0)
Q6. I would recommend closed-loop to others	15 (83)	2 (11)	1 (6)	0 (0)	0 (0)
*Values are n (%)*					
Q7. What did you like about the closed-loop system? Quality of life improved dramatically, I was less stressed thinking about my BMs and diabetes Being more relaxed about my control Always felt safe and in control, always being able to check BG, knowing the HCP could see BGs It was very accurate, easy to use, mobile app was really user friendly Not having to worry about checking my sugars and living a better life The amazing difference it had on my mood and quality of life. Diabetes worked around my life rather than my life working around the diabetes. It made me feel “normal” - very clever system Not having to do BG and then give the correct insulin Not having to carb count or feel guilty about miscalculations/forgetting to administer a bolus Freedom to eat without stressing over carbohydrate counting particularly in social situations. More time in target glucose range. Not having to think about carbs. Freedom of less to have to carry with me. Not having to tell the pump how many carbs I was eating. Being able to eat without thinking The control it gave me during the night while I was sleeping I stopped worrying about overnight hypos and also I became more confident about not needing to plan driving or boost my sugar “just in case” as it became clear I was staying within range The automation, less stressful Freedom of thought, ability to eat at restaurants, reduction of burnout/depression I liked the lack of intervention necessary to keep my bloods in range Having a normal life back/space to think about other things, not just diabetes. Ability to set glucose target to avoid multiple lows. Reactive to diet, exercise, stress - waking reliably to target every morning It required little time/input to get good results, helped reduce my HbA1c. I liked the compact/discrete pump system, liked the app graph display showing CGM data/insulin on phone so can keep pump hidden. It was good to not need finger-stick blood tests					
Q8. What are the things you did not like about the system? Carrying round the extra phone Carrying round the extra phone No low reservoir warning in app, only reminder to refill, no battery low warning in app for pump How much insulin I used In my case my BG rose quite quickly after breakfast and it didn't give me enough insulin. When my BGs were dropping and dramatically it didn't switch off quickly even with ease off. It kept blocking Not a big fan of cannula pumps but not as bad as I thought Not accurately adjusting insulin levels to combat high or low glucose. Choice of pump is not one I would ever consider even for closed loop The system felt like it was a prototype - very noisy, quite a faff to change sets/insulin, etc. Also found sugar went quite high after eating The quality of the pump and infusion sets Initially I felt I had lost control of my diabetes and I could only watch as it swung between high and low. This settled down over the first two weeks At times it didn't catch a high or a low quick enough Boost/Ease off - how long on each based on carb size? Slow to adapt to new routine, frequent morning hypos as a result The insulin was painful at the infusion site and the tethered system I found cumbersome Sudden battery failure on pump, especially at night. Carrying extra phone Pump batteries would die quickly with little warning. Hypos required more carbs to treat compared to regular pump. Blood glucose would generally go quite high with food then sometimes followed by a moderate low					
Q9. Would you like the closed-loop system to have additional features? If yes, which? The needle which goes in the body did not stick very well - stickier would be better Easier set changes To be able to work with smart watch to see current BG and trend Better pump battery, not lithium and a switch off feature when it has an occlusion as embarrassing if some meeting and you can't switch that noise off without doing a complete set change. Better screen to see what pump is telling you Smaller pump would be more discrete Ability to override insulin dosing with more than just boost or ease off options as the algorithm would often still give insulin when below my target glucose Simpler way to change insulin. To be compatible with an iPhone. To be able to switch off the urgent low alert for theatre trips/school plays, etc. Faster acting insulin, more pump support, ability to inject for carbs over time when telling the app a carb count Ability to prewarn of food without carb counting - to reduce postmeal spikes					

BG, blood glucose; BM, blood glucose; HCP, Health Care Practitioner.

There was no significant difference in hypoglycemia confidence or diabetes distress during the fully closed-loop period compared with the usual care period. Results of the INsulin Dosing Systems: Perceptions, Ideas, Reflections, and Expectations (INSPIRE) questionnaire indicate high fully closed-loop participant satisfaction (Table 8).

**Table 8.** Questionnaire Scores

	Closed loop	Control	P
Hypoglycemia Confidence Scale	(*n* = 16) 27.5 (25.3, 35.0)	(*n* = 13) 29.0 (26.5, 35.0)	0.67
Problem areas in diabetes	(*n* = 17) 13.2 (22.5, 48.8)	(*n* = 15) 15.0 (32.5, 51.3)	0.16
INSPIRE	(*n* = 18) 91.1 (77.5, 98.8)		

Data are presented as median (IQR). Values were winsorized at the 10th and 90th percentiles before statistical analysis with paired sample *t*-test.

IQR, interquartile range.

## Discussion

To our knowledge, this is the first psychosocial evaluation of a fully automated closed-loop insulin delivery system. Interviewees universally reported a reduction in diabetes burden describing a sense of “normalcy,” “liberation,” and being “on holiday from diabetes.” They described improved mood and quality-of-life benefits, including reduced worry around mealtimes, improved sleep, and freed-up mental capacity for other priorities.

Although several of these quality-of-life impacts including “time off” from diabetes demands and improved sleep have been reported for commercially available hybrid closed-loop systems,^3^ users highlighted how much they enjoyed the added benefit of not having to carbohydrate count and bolus, and the greater freedom this offered around mealtimes. One unintended consequence noted was that fully closed-loop technology could perhaps lead to unhealthier eating habits (increased snacking and less consideration of carbohydrate intake). This has also been reported with hybrid closed-loop systems,^12^ and suggests individuals might benefit from additional nutritional education when using the system to help promote healthy eating.

In keeping with their expectations, users reported taking a few days to a few weeks to develop trust in the fully closed-loop system. This corresponds with what has been described for hybrid closed-loop systems,^13^ and is important to take into account when planning the timing of transitioning to closed-loop therapy. Warning users that there will be an adjustment period, during which the algorithm will adjust to their insulin requirements and they in turn will build confidence in the system, would be useful to avoid initial worry.

Almost all participants felt the system improved their glucose outcomes; this is consistent with the trial findings where fully closed-loop increased the proportion of time glucose was in target range 3.9–10.0 mmol/L by 13.2% points (mean ± standard deviation 50.0% ± 9.6% with fully closed-loop vs. 36.2% ± 12.2% with usual care; *P* < 0.001).^7^ Although 50% is still below the recommended time in target glucose range,^14^ it is perhaps related to the significant hypoglycemia fear reported by this population, who had high HbA1c levels at baseline. Generally, interviewees seemed satisfied with improved but imperfect postprandial glucose outcomes if it meant reduced diabetes burden and more time to focus on other aspects of their busy lives.

That being said, participants did appreciate opportunities to collaborate with the system through the “Boost” and “Ease Off” function. Some users wanted additional interaction through simplified meal announcements; such systems are being tested clinically by several groups.^15,16^ In contrast, other users preferred the greater freedom from diabetes demands provided by a fully automated system.

Looking forward, an option may be to have the fully closed-loop algorithm as a setting within a hybrid closed-loop system that users could turn on when they were particularly busy with other priorities, burnt out or just needed a break. Perhaps the biggest barrier to this would be the challenges reported around resuming carbohydrate counting and bolusing at the end of the 8 week fully closed-loop period. Although in most cases participants re-skilled quickly, it would be important to adequately support users through the transition period, including providing some refresher education.

Most of the negatives raised by participants related to the insulin pump. We are currently examining the CamAPS HX fully closed-loop algorithm in adolescents with an alternative insulin pump, so the qualitative data from this follow-up study will be informative in terms of hardware burden.

Feedback from the closed-loop experience questionnaires broadly mirrors that from the interviews. The questionnaires are from a slightly larger sample size supporting the generalizability of our findings across the study population. The lack of improvement in diabetes distress and hypoglycemia confidence scores during fully closed loop is surprising given the high closed-loop treatment satisfaction (INSPIRE questionnaire) and quality-of-life benefits reported in interviews. It is possible that improvements may have been seen with a longer duration using the fully closed-loop system; 8 weeks is relatively short, especially factoring in the initial adjustment period.

Limitations of this study include the incompleteness of questionnaire data, as not all participants posted back completed questionnaires. It is possible that those who did complete questionnaires were not representative of the overall study population, and the same may be true of those who consented to be interviewed. Those interviewed were predominantly of white ethnic background and had higher levels of education.

Although this is reflective of the demographics of the overall study population, results may not be generalizable to the wider population with type 1 diabetes. Although the researcher conducting the interviews was not involved in the participants' clinical care, there is still a possibility that participant's responses were influenced by knowing they were part of the same research team.

## Conclusions

This research demonstrates that alongside its glycemic benefits, use of a fully closed-loop insulin delivery system had significant quality-of-life benefits in adults with type 1 diabetes and suboptimal glucose outcomes. These included providing a welcome break from the day-to-day demands of living with diabetes, reduced worry around mealtimes, and better sleep. Drawing on participant feedback and interview responses, we have also identified ways in which the technology could be refined, and education tailored to optimize use.
